# Theoretical and Practical Treatise on Derivation as a Remedy for Some of the Most Common Affections, Such as Plethora, Inflammation, Hemorrhage, &c.

**Published:** 1839-01

**Authors:** 


### ART. II.

1. *Traité théorique et pratique de la Derivation contre les Affections les plus communes en général, telle que la Plethore, l'Inflammation, l'Hemorrhagie, &c. &c.* Par L. F. GONDRET, Docteur en Médecine de la Faculté de Paris, &c.—*Paris*, 1837. 8vo. pp. 328.

*Theoretical and Practical Treatise on Derivation as a Remedy for some of the most common Affections, such as Plethora, Inflammation, Hemorrhage, &c.* By L. F. GONDRET, Doctor of Medicine of the Faculty of Paris, &c.—*Paris*, 1837. 8vo. pp. 328.

2. *Counter-Irritation, its Principles and Practice, illustrated by one hundred Cases of the most painful and important Diseases effectually cured by external Applications.* By A. B. GRANVILLE, M.D. F.R.S., &c.—*London*, 1838. 8vo. pp. 360.

3. *Counter-action viewed as a Means of Cure, with Remarks on the Uses of the Issue.* By JOHN EPPS, M.D. &c.—*London*, 1832. 8vo. pp. 69.

IT has been remarked by M. Louis "that we do not know the therapeutic value of blisters, and that it ought to be studied with the assistance of numerous and well-established facts, absolutely as if we knew nothing regarding them."* Till a recent period this observation might, in this country at least, be extended with a considerable degree of truth beyond the one form of counter-irritation to which it was applied; for, although the expression was in frequent use, though the practice was one most familiar to surgeons and physicians, and various means of accomplishing it constituted important parts of the treatment adopted in the majority of cases with which medical works abounded, we could scarcely be considered as possessing in our language a treatise specially dedicated to this important branch of therapeutics. From 1754, the period of the publication of Dr. Giles Watt's work on Derivation and Revulsion, a work now become antiquated, to 1822, when Dr. Jenner published his letter to Dr. Parry on Artificial Eruptions, no British book on this subject of any mark or likelihood appeared, whilst from the presses of Germany, Italy, and especially of France, the subject received that ample attention which its importance merited. As a consequence, apparently, of this superior attention, we have remarked that continental practitioners employ a greater variety of remedies of this class, and repose more confidence on them, than those of this country.

These observations are calculated to show that the British works at the head of this article are by no means superfluities in our literature, if they are really fitted to supply the deficiency which we have seen to exist. Before, however, proceeding to examine into this point, it appears incumbent upon us to give such a general view of the subject as may place the reader himself in a position to judge of the respective merits of these works, and likewise to clear it from certain sources of confusion

* Recherches sur les Effets de la Saignée en quelques Maladies Inflammatoires. Par P. Ch. A. Louis.

and error, which a careful perusal of them, and, in the deficiency of our own literature, of various foreign publications, has shown to exist.

I. The terms employed, it will be observed, in this country and on the continent, to express the action produced by these remedies, and the remedies themselves, are different. In England we now speak of counter-irritation and counter-irritants; Dr. Epps's expression is counter-action: abroad, the terms are derivation and derivatives, and revulsion and revulsives, which same terms were anciently used in our own country. It is a reasonable question whether terms so dissimilar do in fact signify the same thing; and, whether they do so or not, what they do signify. That the word *derivation*, and its correlative *derivative*, have a sufficiently wide sense, is evident from passages of the work of M. Gondret; for he says that "normal derivation consists in the regular course of the different evacuations proper to the human body, cutaneous and pulmonary exhalations, urine, and fæces. Therapeutic derivation has for its object to reestablish normal derivation, when it is retarded or suspended." (p. 13.) Dr. Epps occasionally employs his word *counter-action* in a sense equally extensive; for he remarks, after describing the cold and hot stages of ague, "All pass away on the occurrence of the perspiration; and what is this perspiration but a counter-action set up upon the skin, and thus relieving the internal disease?" The same writer remarks that, "in cases of abdominal dropsy, violent purging is induced to excite counter-action, in order to remove the dropsy, and at the same time to overcome the action connected with the effusion of a dropsical fluid. Indeed, in many other diseased states, counter-action is frequently excited by the internal administration of medicines." (p. 56.) Definitions, or rather descriptions, like these, give to the words employed in the titles of these works a meaning co-extensive with the whole method of cure adopted in hyperemia and inflammation, whether applied to the surface or administered internally; and an essay on derivation would be a treatise of therapeutics. When we examine, however, the application of the terms these writers respectively use to the means they actually employ, and to the direct effect produced by these means, we find that counter-action, counter-irritation, and derivation, counter-irritants and derivatives, have the same meaning; the former words describing a degree of irritation of the surface, with an afflux of blood to it, and in most cases an increased secretion from it; the latter, the means by which such effects are produced. All the words are hypothetical, (that employed by Dr. Epps excepted;) derivation, and its correlative, implying that the afflux of fluid to the part irritated is the means of cure; counter-irritation, that the irritation is so. This is, however, immaterial; as is likewise the etymological sense of the words, (a point on which Dr. Granville lays needless stress,) provided it is agreed to limit them to express the fact that, from the application of substances capable of producing such immediate effects on the surface, diseases seated elsewhere are relieved or cured; a fact which experience only can teach us, though an analogy which will be subsequently adverted to may have suggested their trial. Revulsion and revulsive have, at least since the days of Wiseman, been understood to differ from derivation and derivative only in degree. Leaving the humoral pathology involved in all the words out of the question, the former express that the remedies applied, though of the same nature, are more violent, and are applied at a greater distance from the part morbidly affected than when the latter terms are employed.*

It will thus be perceived that there was a source of confusion arising from the employment of different words to express the same thing; this difference arising from the medical doctrine reigning at the time the terms were introduced, and their adaptation to such doctrines: the contemporaries of a humoral pathology ascribing the cure to an afflux of humours to the part, the solidists to its irritation. Dr. Granville has dedicated eight pages of very small scholarship to a further confounding of the original confusion. Besides showing a singular want of skill in discussing what he considers the three modes of action displayed by these remedies prior to his *discoveries*, he introduces a fourth element of confusion in the following very luminous terms:

"One of the external applications to which I allude I have found to produce four very distinct and well-marked actions; or, if the reader pleases, four consecutive degrees or varieties of the same action. Three of these are identical with the three modes of action already described, (counter-irritation, revulsion, derivation;) the other, or the principal mode, judging only by its manifestation when employed, and by its rapid success, is not comparable to any of them. It stands alone: I believe it never to have been obtained before by the ordinary means of external medication; and its influence on the nervous system admits of only two plausible solutions: that of a *shock*, (dissimilar, however, in every respect to that of electricity or electro-magnetism,) and that of a rapid absorption of the substance employed. It is not possible to explain what takes place under the almost magic influence of the application alluded to, in all cases of acute pain of the nerves, of spasm, of nervous head-ach, and of very intense tooth-ach, unless we adopt one or the other of the preceding solutions." (p. 7.)

II. Those natural analogies by which counter-irritation was probably suggested in the infancy of our art, and from which its adoption receives illustration and authority in the present day, are of too much practical importance to be passed without notice. All the writers before us mention them. Dr. Granville says, "If, in a case of gout affecting the stomach, we succeed in relieving that important organ by means of counter-irritants applied to the instep or the toes, we do so because we have often witnessed how nature quickly expels a dangerous attack of gout from the stomach by the appearance of a well-defined, red, tense, and exquisitely painful swelling of the foot." Dr. Epps treats this branch of the subject at considerable length; and we consider his first chapter as a valuable record of examples drawn from sources ancient and modern, and from his own experience, of the danger accruing to patients from the suppression of chronic eruptions and ulcers; though we must be allowed to remark that, by suitable constitutional treatment, medicinal and dietetic, the patients might have been relieved from a loathsome local affection, without danger to life, much more frequently than Dr. Epps seems to imagine; and we are somewhat surprised that an experienced physician should have apparently counselled rather a slothful acquiescence in disease than an adoption of measures for the restora-

* Derivation differs from revulsion only in the measure of the distance and the force of the medicines used: if we draw it to some very remote, or it may be contrary, part, we call that revulsion; if only to some neighbouring place, and by gentle means, we call it derivation.—*Wiseman's Surgery.*

tion of *actual* health. These eruptions, from the crusta lactea of infancy to the acnes and impetigines of adult or even advanced age, are very properly regarded as the safety-valves of the constitution; but neither reasoning nor experience counsels us against dealing with them through the constitutional errors on which they are dependent. Would not the correction of faults of regimen and the prudent employment of remedies for plethora, conjointly with the local treatment for the ulcer existing in the following case, have produced a result much more creditable to our art, and beneficial to the patient, than that which actually occurred? We give the case in Dr. Epps's own words, which most people will regard as more distinguished for point than reverence.

"I remember a dean who made 'a god of his belly,' and who every morning was visited by his medical practitioner on account of an ulcerated leg, with which he was, he said, 'afflicted;' the medical practitioner thought 'blessed.' Becoming tired of the constant torment of this ulcerated extremity, and being at the same time, perhaps, unwilling to pay the constantly repeated fees, the luxurious dean went to a surgeon of considerable eminence for the cure of diseased legs. The leg was healed, and the chapter was left vacant; for the worthy member who had filled a place in it so many years, with so little benefit to the public and with so much ease to himself, was absent: in other words, he died; and apoplexy was death's messenger.

"The dean's medical adviser had recommended him not to have the leg healed; for, as the dean would eat, and, as the poet says, 'would sleep,' it was the conviction of the practitioner that some drain must exist in the system.

"The dean, however, was not satisfied. The leg-doctor was not of the most accurate or of the most extensive knowledge: he did not see the principle of the treatment. The patient came wishing to have his leg healed: the object was effected. The leg-doctor was praised; but the dean, as was before stated, soon ceased to give his recommendatory statements of the man's skill." (p. 6.)

In the same part of the work we find recorded the important fact that in families, some of whom labour under insanity, and others are exempt from this malady till the day of their death, the latter will often be found to have had through life scrofulous ulceration in the leg, neck, or some other part of the body. Indeed, the analogies drawn from natural disease in favour of counter-irritation are in general very interesting; but some we think strained beyond the limits which strict reasoning justifies. In this category those appear to us which are drawn from the exanthemata; the author regarding the eruption of small-pox as a counter-irritation established by nature for the relief of epigastric pain, vomiting, and fever; whilst that of measles is similarly destined to remove the catarrhal and febrile symptoms of the complaint. This view appears to be rather assumed than proved. A considerable mitigation of the internal distress in these two diseases generally accompanies the eruption; but it occurs on its first appearance, before the inflammation of the skin exists to an extent to act as a counter-irritant. Indeed, their whole progress wears more the aspect of the disease fulfilling its destined periods, than that one of its parts is the remedy of the other. If we attempt to extend the analogy to scarlatina, it fails; for here great hyperemia of the skin, so far from relieving an ardent fever and great internal oppression, is associated with them throughout the disease. The deadly nature of certain cases of the exanthemata, in which the eruption fails to occur, cannot be advanced with any confidence to confirm the views of the author; for it is at least as reasonable to attribute this failure in the ordinary course of the disease to its inherent deadliness, as to suppose, with Dr. Epps, the causation to take place in the reverse order.

III. Besides the suggestion and confirmation of the practice now under consideration, derived from these natural analogies, accidents or surgical operations not performed for the purpose of exciting counter-irritation, may, in the course of their cure, produce an effect on some previously existing disease, well fitted to illustrate the therapeutic value of intentional counter-irritation, and even the relative utility of different modes of effecting it. Dr. Epps's pamphlet is rich in cases belonging to both these classes. The following example of accidental counter-irritation appears very important and instructive:—Captain Innis was tapped, and nineteen pints of water were drawn off. The abdomen began again to fill, and several pints of water had reaccumulated. Some boiling water was spilled on the captain's leg. Inflammation came on, an ulcer was formed, and spread. In proportion as it spread, the accumulation of water in the abdomen diminished, and at length the officer was perfectly cured of his dropsical affection.

We think that some exception might be taken to a proportion of the author's cases, which fall under one or the other of these heads; for, being rather culled from various writers than derived from his own experience, many of them are deficient in the fulness of detail required for a just estimate of the power of the (accidental or unintentional) remedy. This objection applies to the following case from Richter. This eminent surgeon had extirpated both mammæ for supposed scirrhus. On the fourth day after the operation, an indurated gland was discovered in the arm-pit, suppuration of the wounds appeared on the fifth, and during this, which lasted for five weeks till the breasts were healed, the tumour vanished. We think that a full detail of the case would have shown, either that the axillary swelling had been generated by the irritation of the incision, or that, at all events, it was of the nature of common inflammation, and not carcinomatous; for we are convinced that it does not consist with the experience of any surgeon that tumours of a malignant nature are dispersed by the suppuration induced by the removal of a portion of the diseased structure. Again, Dr. Epps informs us that Brambilla saw phthisis cured by amputation of a leg. We have seen many cases, at least very much resembling phthisis, cured by this means: we did not, however, ascribe this to the counter-action of the operation, but to the patients being relieved from the irritation and drain to their constitution arising from a diseased joint.

IV. We should object to all of the works before us, that they are less systematic treatises on counter-irritation than pleadings in favour of some specific mode of effecting it; but it is only just to remark that, whilst the unpretending pamphlet of Dr. Epps is the least exceptionable on this score, the more voluminous and imposing tome of Dr. Granville is the most so; and to the last-named individual belongs the additional distinction of being the sole advocate of a secret *nostrum*, and this nostrum his own. We should have been gratified to find such questions as the following discussed, and the experience of the writers applied to their solution:—In acute inflammatory disease, how far will counter-irritation supersede bloodletting and other exhausting measures? Should, in such diseases, epispastics be at once associated with the means employed, or should the latter in all cases precede them? In phlogosis of a more chronic character, should depletion enter into the plan of treatment, or may the disease be trusted to counter-irritation alone; and, if the former is to be employed, ought it to precede the latter? These are practical questions, and such as a good deal of observation of what is done and omitted by various medical men in this country, convinces us are not so far settled as to induce an uniformity of practice; and an examination of them, if not a final settlement, might have been reasonably expected in works of the character now under consideration. Another point, too, of much importance is left untouched by these writers, with the exception of Dr. Epps. When irritants are applied to the surface, certain manifest effects are produced there; such as rubefaction, vesication, and cauterization, and, it may be, ulceration with secretion of pus. Besides these effects on circulation and secretion, there is a sense of heat and smarting, arising from an impression on the nerves of the part. According to the choice of the practitioner with regard to the means he employs, or the duration of the application of the same means, certain only of these effects may be produced, others avoided. It is an important question how far one only or more of these forms of effects are essentially connected with the curative influence on disease; and, provided that more than one is so, which is the most influential? The decision of this question, it is obvious, should precede the other, "What counter-irritant is the best ?" and would suffice to settle it; yet, whilst the latter is the main theme of three volumes, the former is mentioned only by Dr. Epps.

We attach the more importance to this writer's opinion on this point, because it is, though not identical, considerably accordant with that of one of the master-spirits of our art, Dr. Jenner. The former gentleman, after eulogizing the efficacy of issues, thus expresses himself:

"It may be asked, *on what is the counter-action* connected with the issue dependent? Upon the formation of the pus, and the *state* of the surrounding parts connected with its formation. The formation of pus necessarily implies a new action in the vessels of the part. The vessels surrounding the part where this action is going on are brought into a dilated condition. They are thus predisposed to be affected by any cause producing an irregularity in the distribution of the blood; and thus, the diseased action being induced in them, it is prevented taking place in any internal part where a debilitated state of the parts may exist." (p. 64.)

Dr. Epps meets the reasoning deduced from the small quantity of matter formed by the following facts:

"A. B., a young gentleman of high moral character, aged twenty-one, had been troubled for several years by a small transparent drop of viscid lymph being continually at the termination of one of the passages of the body. It was no trouble to him; but when, from any cause, it was dried up or was not formed, A. B. was seized with the most intolerable headaches, not relieved until the discharge was produced." (p. 64.)

Dr. Jenner, in his letter to Dr. Parry on Artificial Eruptions, insists, in common with Dr. Epps, on the necessity for the production of a fluid. He says that he found, in the case of artificial eruptions produced by the ointment of emetic tartar, "that the relief afforded was almost invariably timed, not simply by the first blush of inflammation, but by the appearance of vesicles which contain a *secreted fluid;* and that these seem absolutely necessary for the production of constitutional influence." Again he remarks, (and it is not unpleasing to trace the influence of his researches on cow-pock in his reasoning,) that "a vesicular eruption, in general, seems the favorite scheme of nature for limiting the duration of peculiar morbid actions." "Every pimple with a vesiculated head has an errand to perform for the benefit of the constitution." Among the subjects connected with counter-irritation and artificial eruptions, suggested by this distinguished individual for further elucidation, is the following: "How far the peculiar constitutional influence results from the *eruptive* form of the local irritation excited, and is analogous with the laws of *secretion*." It is evident that, though one of these writers suggests the employment of the ulcerative, the other that of the vesicular or pustular form of irritation, both regard the secretion induced as the efficient instrument of cure.

V. With the exception of Dr. Epps, the writers before us display little of the analytical spirit in which we should have been glad to see the whole subject treated: they furnish, however, much information as to the influence of certain counter-irritants on different diseases; and the substance of this information we shall endeavour to impart to the reader in as systematic a form as we can invest it with.

I. *Heat.* Heat is an agent which receives much attention from two of the writers; and, as its powers are less generally known and appreciated in this country than abroad, we are glad of an opportunity of exciting attention to it. The illustration, by Dr. Epps, of intentional counter-irritation derived from the accidental application of heat, has been already mentioned. The same writer gives us some valuable information regarding the intentional employment of the same agent. The following examples of recovery from poisoning are very valuable. A gentleman took ten grains of opium, and in about an hour after was found by his medical attendant in an apoplectic state. The stomach-pump was then unknown, and, the stomach being overpowered by the poison, tartar emetic and white vitriol failed to produce vomiting. Hot water was applied to the thighs, legs, and arms alternately for twenty-four hours, when the patient recovered; the inflammation in the scalded parts being afterwards so severe as not to allow the patient to touch them with his clothes. It is probable that the practice which here proved so successful might be extended to other cases of apoplexy besides those from poisoning. In the other case, in which a blacksmith had taken "a *quantity* of prussic acid," boiling water was poured over the legs without any effect. The legs were then scarified in numerous places, and another quantity of boiling water was poured over the limbs thus scarified. The first sign of restoration was a slight spasmodic contraction of the muscles, and in a short time spasmodic action took place in many parts of the body. The pulsation of the heart and the respiratory motions were restored, but weakly and irregularly. In a quarter of an hour the legs began to swell, and there was general excitement, but no pain. There was progressive improvement, which is minutely described; but it is sufficient for us to say, that on the evening of the second day he became quite sensible, and was ultimately restored to perfect health. We think this case creditable to the practitioner. Cold affusion might possibly have produced the same result more speedily, and certainly without subsequent suffering; but we are not willing to intimate that any proceeding would have been preferable to one which produced recovery under such deplorable circumstances as those described.

Fomentations are too generally prized, and the correct mode of applying them is too familiar, to render it needful to transcribe Dr. Epps's testimony in their favour, or his minute description of the best mode of employing them; but the following statement transcends any idea we had formed of their efficacy, and we regret that the author does not confirm it from his personal experience.

"Dr. Wigton, of Edinburgh, states [where?] that by fomentations he always succeeds in curing *puerperal inflammation*, if called before the disease has proceeded to the last stage. The pain, let it be remembered, is often increased by the two or three first applications, but afterwards the pain diminishes. The rule of Dr. Wigton is, *never to desist till the pain is relieved*, yea, *removed*. Perseverance is always necessary; no fixed time for the continuance of the fomentation can be named: *till the pain is relieved* ought to be the criterion." (*Epps*, p. 27-8.)

M. Gondret dedicates a chapter of considerable length to the consideration of the same agent, heat. It opens with the well-known aphorism of Hippocrates: "Diseases which medicine does not cure, steel cures; those which steel does not cure, fire cures; and those which fire does not cure must be regarded as incurable." Supported by this high authority, M. Gondret extended his historical researches from the 80th Olympiad to the present era, and found that all distinguished physicians had given their testimony in favour of this remedy. He proceeded to its practical application, and after finding that phthisis, in a certain stage, yielded to its power, he assailed with it palsy, apoplexy, epilepsy, rickets, cancerous tumours, gout, neuralgia, visceral enlargements, cataract, and amaurosis. His experience in these diseases has led him to regard fire as the tonic "*par excellence*," and, as it reestablishes at the same time the physical and moral faculties, the regulator of the vital principle.

These flourishes suggested no pleasing presentiments of our further progress through the work; but we find the facts detailed of greater value than we anticipated. Those relative to sincipital combustion,—in other words, burning the crown of the head with a metal heated to a white heat,—in certain affections of the sensorial and motor systems, appear to us very interesting and important. A brief abstract of one or two cases will, we think, lead the reader to concur with us in opinion.—A girl, sixteen years of age, subject from her third year to convulsive movements of the muscles of the neck and momentary loss of consciousness, became, when twelve years old, decidedly epileptic, so that she had monthly from fifteen to twenty fits of this disease. Her intellect was obscured, her appearance idiotic, her movements were restrained and slow, and her step was tottering. Bleeding and antispasmodics were employed in vain. On the 27th March, 1816, sincipital combustion was performed, and there was no fit till the 16th April. On the 8th of May, a thin scale of bone exfoliated from the cauterized part. On the 10th of this month, the cautery was repeated above the occipital protuberance. From the 27th of March to the 6th of October, a period which, prior to the cauterization, would have furnished more than a hundred fits, there were but three, and those very slight ones; whilst the intellectual faculties were much improved, and the movements became free and lively. Seven months after the first use of the cautery, this patient married, and a year after bore a healthy child.—In the following case of double cataract, the effect of the same remedy would appear to have been very beneficial. A sergeant had inflammation of the right eye, with cephalalgia, and was sent to consult M. Gondret. On the 8th of December, 1824, his state is thus described: Right eye, vision null, conjunctiva inflamed, with a degree of puffing resembling chemosis; no pain; pupil motionless and contracted; cataract very well marked: left eye, sight constantly interrupted by a thick mist, and at intervals entirely confused; evident opacity of the lens, which is of a grey-white colour. On the 9th of December he was cupped in the nape, and the crown of the head was cauterized. Passing over other reports, we reach that of August, 1825, which is as follows: The cataract of the left eye is almost invisible, and the vision good; in the right eye, the conjunctiva is cured, the pupil is still contracted, but possesses some motion; the lens is very visible, though less opaque and less dense than it was. The sight of this eye, which formerly was null, is sufficiently reestablished to enable the patient to distinguish the features of any one, especially when he looks in the direction of the small angle of the eye, where the crystalline has the least opacity. In September, the vision of the left eye was well preserved, whilst that of the right was improving.

This memoir of the author on the utility of fire in medicine is the subject of a report by the commissioners, MM. Portal, Thénard, and Percy, deputed by the Royal Academy of Sciences to enquire into its merits. This report is in the highest degree favorable to the disinterestedness, modesty, and integrity (*loyauté*) of the author, and to the efficacy of the means he recommends. They declare that the application of fire in general, and in particular the burning of the head, are exempt from danger, when employed by one possessing the requisite pyrotechnic skill; and that they themselves had witnessed, on five important (*majeures*) occasions, where physicians, wearied with the obstinacy of the disease, had abandoned the patients for ever, sincipital cauterization, performed by M. Gondret, produce changes the most astonishing and the most salutary.

It has long been our opinion that, in affections of the brain and nervous system, counter-irritation of the scalp is less frequently and decidedly employed in this country than it deserves to be. As is the case with many peculiarities of British practice, this chariness can be traced, we believe, to the influence of one successful writer. Dr. Cheyne, in his work on Hydrocephalus acutus, published nearly a quarter of a century ago, reprehended the practice of blistering the scalp in this and other cerebral affections; and his counsel has not only been adopted, but extended beyond the circumstances to which he applied it. We find even the present writer, M. Gondret, in a tone singularly inconsistent with the general tenor of his work, decrying *leeches* and blisters to the scalp in acute affections of the head, and asking "who has not seen cerebral and ocular inflammation augmented under the influence of leeches, applied alternately on the temples and round the eyes and ears?" With the single exception of the application of these animals so near the eye that the irritation caused by their bites extends to the inflamed organ, we have not only not seen that happen which is implied in

M. Gondret's query: but we have observed the very reverse,—relief of cerebral and ocular inflammation. We may remark, that a practice very analogous to the sincipital burning of our author, the application of caustic potash to the crown of the head, is much employed in diseases of the eyes by some of the ablest oculists in this country,—among others, by Mr. Guthrie; but we are not aware that this practice, less alarming to the feelings of our countrymen than the fiery proceedings of our Gallic neighbours, has, by a very natural analogy, been extended to diseases of the brain.

2. M. Gondret was naturally desirous of obtaining a means of effecting the surface to an extent sufficient for producing an efficient counter-irritation, in a manner less shocking to the prejudices of some physicians and less painful to the feelings of patients, than combustion in general, and sincipital combustion especially, are found to be. This means he considers that he has obtained in his "*pommade ammoniacale*," the preparation of which is thus described: Take of hog's lard seven drachms, of oil of sweet almonds one drachm and a half, and of liquid ammonia (of twenty-five degrees) from five to six drachms. Melt the hog's lard, mix with it the oil, and pour them into a wide-mouthed bottle with a ground-glass stopper; then add the ammonia, close the bottle, mix the contents together by shaking, and keep the mixture in a cool place.

The evidence adduced by the author of the efficacy of this application, not merely in those chronic cases to which, prior to its adoption, he would have thought his heated iron suitable, but in acute inflammatory diseases, in which blisters are the counter-irritants ordinarily employed, is most decisive. Regarding its influence over this latter class of diseases he thus expresses himself:

"With this liniment, a few minutes suffice for its good effect. The disease, it is true, is not always entirely cured: its intensity only is abated, and it is on this account so much the more easily managed, and, under suitable treatment, the result is almost always fortunate. Even in inflammations of a considerable degree of severity, the disease, being attacked at first by the vesicating liniment, miscarried as it were, and appeared to be reduced to the mere artificial inflammation of the dermis. Even notwithstanding previous attacks of the lungs, the disease runs a mild course, and terminates from the sixth to the tenth or eleventh day. I can speak as confidently regarding the inflammation of many other tissues, such as the mucous and serous membranes, the fibrous tissue, &c. The same application produces the same results. An excellent use may be made of this remedy in the different forms of angina; in croup, for instance. The rapid action of the vesicating liniment has another advantage, easy to be perceived,—the physician can order the application of the remedy, and immediately know its effects. The phenomena which he observes enlighten his diagnosis, and make him acquainted with the real indications of cure. This is the proceeding which I constantly pursue, and I cannot express the satisfaction I feel in having adopted it." (*Gondret*, p. 56.)

The author's experience of the effect of his "pommade ammoniacale" was, like his observations on actual cautery, the subject of a memoir to the Royal Academy of Sciences, and of a report from the commissioners of that learned body, MM. Portal, Hallé, and Percy. They express themselves strongly in favour of the remedy. It is much more prompt in its action, they say, than cantharides, exempt from the distress occasioned by the absorption of this medicine, and capable of much more varied effects. If the skin is to be excited, perspiration reestablished, and some subcutaneous engorgement to be dissipated, light and hasty frictions accomplish these objects. If a rubefacient effect is sought, its application for one or two minutes, spread thickly on linen, answers the purpose. In case vesication is required, a similar application for five or ten minutes produces the effect. On the other hand, should absolute cauterization be sought without alarming the timidity of patients or the prejudices of certain medical men against the use of fire, a somewhat longer application attains this end, so desirable in many neuralgias.

This ammoniated preparation leads us to the consideration of certain washes recommended to public notice by Dr. Granville. The analogy which suggests the transition consists in the facts that, whilst ammonia is the avowed basis of M. Gondret's preparation, the same alkali is at least an ingredient in the washes of Dr. Granville, and, so far as we can decipher his Sibylline leaves, an important ingredient. We would say, moreover, that, on comparing the candid statement of M. Gondret of the effect produced by his pommade, and that of Dr. Granville regarding his "antidynous lotions," divesting the latter, as far as we can, of "shocks (dissimilar, however, in every respect to that of electricity or electro-magnetism,)" and other mystic matters, we find the greatest possible resemblance between the effects attributed by the writers to their respective remedies. Here, however, the parallel between the two authors ceases; for, whilst nothing can exceed the openness and candour of the Frenchman, and whilst we cordially concur in the praises bestowed by the commissioners of the Royal Academy of Sciences on his disinterestedness, modesty, and "*loyauté*," we have not been impressed with any signal display of these qualities in the work of Dr. Granville.

A few parallel passages from both authors descriptive of the effect of their remedies will, we think, show that their nature is essentially the same.

With regard to the promptitude with which pain is allayed by his liniment, M. Gondret describes a very acute rheumatism of the posterior muscles of the neck under which M. Renauldin, principal physician of the hospital Beaujon, laboured.

"A small wound was made over the most painful point. When the pain raged with intensity, M. Renauldin requested that the liniment should be applied to the wound itself. This was done and, instead of increasing the pain, it imparted great relief. M. R. declared, *during the action of the pommade, he experienced only pleasure on account of the cessation of the pain.* With the liniment I recommend, *a few minutes* suffice to produce this good effect." (*Gondret*, p. 55-6.)

"I found that without charring the skin or producing an eschar, such combinations would on a mere application to the external surface of the body, give rise to peculiarly energetic effects on the disease, *in the brief space of a very few minutes* (sometimes seconds only.) As the nature of the first impression in all cases of pain was ascertained to be an instantaneous removal of the pain itself, even where no other phenomenon was required or permitted to arise from any of the applications in question, I gave to this class of counter-irritants the name of antidynous." (*Granville*, p. 51.)

"Others *appear to consider the artificial pain as a relief, and a few even have hailed that pain as a pleasure.*" (*Ibid.* p. 86.)

Both rapidly blister.

"Do we require for any reason a vesicating effect? It is sufficient to leave the liniment applied for *a period varying from five to ten minutes*, and the physician before quitting the patient can see the effect of the remedy which he caused to be applied when he entered, an advantage which *boiling water* or boiling oil could produce more quickly, but *too abruptly, too painfully,* and too irregularly, to entitle them to the preference." (*Report to the Royal Academy of Sciences; Gondret,* p. 45.)

"But the relief of pain, in a manner almost magically rapid, is not the only phenomenon produced by antidynous or counter-irritating lotions. Another very striking characteristic of them is that of raising, if necessary, *in a few minutes,* a complete and genuine blister, equal to that produced by the best blistering ointment after several hours' application, or by *scalding water,* but accompanied by *pain much less intense in degree,* and much shorter in duration." (*Granville,* p. 53.)

Both, besides blistering, produce a rubefacial or cauterizing effect according to the length of time they are applied.

"Do we wish to produce rubefaction, we apply a portion of this *pommade* one or two lines in thickness spread on linen, for one or two minutes. . . . Is it necessary to imitate the cauterizing action of fire, we shall succeed by prolonging the application." (*Report to the Royal Academy of Sciences; Gondret,* p. 45.)

"Without necessarily producing rubefaction and cauterization, although, if sufficient time for the purpose were allowed, the same combinations would produce the latter phenomena also." (*Granville,* p. 51.)

We shall notice only one coincidence more, regarding the principle on which both these applications relieve pain.

"Taking as a guide of practice this sentence of Hippocrates: of two pains formed simultaneously, but in different parts, the stronger obscures the other; if an internal organ is affected with a pain arising from an inflammation or an arthritic or neuralgic metastasis, in an instant it will be easy to lessen it or to cause it to disappear, by opposing to the prevailing pain a new pain. In these cases, the remedy cannot be applied with too great promptitude." (*Gondret,* p. 54.)

"It now remains for me to assert, as a crowning of all this, that so soon as the pain of the lotion is felt, that instant the inward pain, for the removal of which it was applied, is suspended and at last vanishes." (*Granville,* p. 88.)

It would be easy, but superfluous, to multiply quotations to prove that the immediate effects of these two agents are identical; and when we compare the diseases in which they are employed by their respective authors we find that the same ultimate effects result from them, in other words, that they remedy the same complaints. From these facts and the statements of the authors, the inference seems inevitable, that both individuals are employing the same agent, ammonia, in a more concentrated state than it has hitherto been usual to employ it, either as volatile liniment or in any other way, and that the sole difference between them consists in the one having selected an unctuous or saponaceous vehicle, whilst the other diffuses his ammonia through "liquid odours." With regard to discovery, M. Gondret does not claim any beyond the application of a counter-irritant familiar to the profession at least since the days of Sir John Pringle, in a state of concentration sufficient to accomplish objects not formerly expected from it, and in a convenient form for application. Neither does Dr. Granville's claim of originality rest on the employment of *ammonia;* on the contrary, his 24th case was one of epilepsy observed by him in the *Hôpital de la Pitié* in 1816 and 1817, and cured under the care of M. Serres by means of a cerate composed of purified butter of cacao and liquid ammonia applied over and along the spine, by which vesication and a discharge were produced. Now, the commissioners of the Royal Academy of Sciences state that M. Gondret sometimes substitutes this very butter of cacao for his ordinary unctuous vehicle, six drachms of the former replacing an ounce of the latter. M. Gondret, be it remarked, is an old labourer in the field of counter-irritation, and though his book does not furnish positive evidence of it, for he does not stickle for priority and does not often insert complete dates, we think it probable that the counter-irritant employed by M. Serres had been originally suggested by him. On this point, however, in the absence of positive evidence we have no right to insist, nor is it necessary that we should. But we have proof, even in the work before us, that M. Gondret used his "pommade ammoniacale" as early as 1821, (see p. 35,) whilst the date of the first application of Dr. Granville's lotion is eight years later. M. Gondret's memoirs to the Royal Academy of Sciences must have been printed as early as 1818, at least; as the first edition of his work, *sur le Feu,* to which the Academy's Report is attached, was published in the end of that year or beginning of 1819. Even the present work was published a year before that of Dr. Granville. Considering these circumstances,—considering, moreover, how ardent a cultivator of medical literature Dr. Granville has ever shown himself,—we cannot help feeling some surprise that the labours of M. Gondret should have entirely escaped his notice; especially that that book should have done so, of a portion of which (that relative to the "pommade ammoniacale,") his own wears the appearance of being a mere amplification.

Whether the coincidences of thought and language which have struck us in perusing the two works and of which our readers have been furnished with a few specimens, arise from the one book having formed the foundation of the other, or whether they are only examples of that accordance of opinion which two individuals engaged, unknown to each other, in the investigation of the same subject may be supposed to display, Dr. Granville alone can positively determine. With regard to our own opinion on this point, besides that we are wishful to acquit Dr. Granville of plagiarism, finding, after making the reasonable deductions from the extraordinary verbosity and inflation of his style, that nothing is predicated of the one preparation, that is not declared to be true of the other, we are firmly of opinion that the agents employed by these two writers, are in all essential respects the same, and that Dr. Granville was labouring under some extraordinary delusion when he fancied he had made "*discoveries.*"

We think that the evidence adduced by M. Gondret shows that strong liquid ammonia is a very efficacious instrument of counter-irritation, capable by slight varieties of management of more varied effects on the surface, and consequently on disease, than most of those with which we are acquainted, and that, whilst it is capable of doing much good, it is difficult to conceive that any evil can be produced by it, excepting from a want of judgment in its application which it is scarcely possible should occur. It would have given us much pleasure to add, that Dr. Granville, whilst he had contributed by the mass of facts he has brought forward to confirm the views of M. Gondret, had written a sensible volume to diffuse the knowledge of a valuable remedy among the profession in our own country. The former praise (he may thank us for it or not) we think is his due; but to the latter he has no claim; for, as we remarked in our last number, he has addressed no work to the profession. Conscious, apparently, of some demerit, he has made an anticipatory appeal from the decision of a competent tribunal, the profession, to that of an incompetent one, the public. This appeal, however, will avail him not: he is before a professional tribunal, and its award he must abide. One tithe of the talent and judgment which on many occasions he has displayed, would have enabled him to write a book on this subject for which he would have been praised and thanked. Some strange mistake, some unaccountable condition of bewilderment has led to the production of one, which will be for a few weeks a byword and a scorn, and then (let us hope) will be forgotten.

3. The subject of the third means of derivation employed by M. Gondret, cupping, is introduced by a description of the effect of atmospheric pressure on the body generally and the various functions, such as respiration and suction which are directly dependent on such pressure, and on those, the circulation and cerebral functions, which receive from it a secondary influence. This physiological account, which will be found between the 83d and 98th pages, does not present any novelty, but it is accurate and might be read, especially by the general reader, with advantage. The following examples of the pathological effect of diminished atmospheric pressure are interesting to all.

"In the month of December, 1747, sudden deaths were very frequent at Pluviers in the Gâtinois, and M. Duhamel observes that in this same month the barometer fell in less than two days one inch and four lines, that is to say, from twenty-eight inches to twenty-six inches and eight lines, which certainly might produce great effects on the living body, since the variation of an inch in the barometer indicates a difference of about a thousand pounds in the weight of the atmosphere."*

"At the summit of the Vosges, wounds and ulcers bleed easily, and the formation of the clot in hemorrhages is difficult; ophthalmias are very obstinate; catarrhal quinsies are very common and difficult of cure; hernias easily become strangulated, and metastases are frequent; pregnant females are subject to difficulty of breathing, and to floodings and miscarriages. It is often necessary to convey patients to the foot of the hills, that they may respire an air less attenuated.† These observations of Saucerotte have been confirmed by those of M. Hippolite Cloquet. M. Dolomien when in feeble health, his chest being especially delicate, having ascended to the *Pic du Midi* (a height of 9,000 feet) experienced speedily extreme weakness, considerable oppression and a spitting of blood, which placed his life in real danger, till he was conveyed down the hill. More robust travellers, however, such as MM. de Humboldt, de Saussure, and others, attained to a higher degree of atmospheric elevation without experiencing such painful effects." (p. 96-97.)

The opinion of the author regarding the effect of atmospheric pressure is not confined to its conditions which are relatively to men abnormal, but he thinks that in a diseased state the ordinary atmospheric pressure is a source of distress.

"When the health is impaired, the weight of the body and consequently that of the atmosphere becomes extremely perceptible. Thus, in cerebral inflammation or plethora, there is often a sense of weight in the head so considerable that the patient cannot raise the part; and it is in the highest degree probable that an organ, the seat of a sanguineous engorgement, cannot form an equilibrium against the pressure of the atmosphere without suffering." (p. 100.)

We are not aware of any facts which lead to the conclusion that the

* Duhamel, Encyclopedie Method.; t. 1. † Saucerotte, Mélanges de Chirurgie.

normal pressure of the atmosphere is injurious to our organs, sound or healthy, and we cannot discover in the circumstance here stated by M. Gondret, any evidence that such is the case. When the brain is the seat of hyperemia or inflammation, the head feels heavy and the act of raising it is painful, but so is that of moving it laterally or rolling it; it is moving the diseased organ, not raising it only, which occasions distress; or, if more distress is experienced in raising it, this arises from the movement itself and the muscular exertion required to effect it in this direction being greater. Lying with the head raised, so far from augmenting the distress, is one of the means employed by medical men to remedy it. The benefits of the revulsion effected by cupping did not require, and certainly could not receive, any confirmation from this vague speculation.

Dry cupping, a remedy strongly recommended by Hippocrates and Celsus, and more recently by Prosper Alpinus, had fallen in modern times into comparative desuetude excepting among the Germans; the author, as it were, resuscitates it, and produces evidence of its being an important agent in alleviating, and, in many cases, removing disease. Affections of the heart and various inflammatory diseases having their seats in the head, chest, epigastrium, and uterus have been removed by it. The mode in which the relief is effected is abundantly simple, and we shall state it in the words of the very favorable report of the Royal Academy of Sciences on this part of M. Gondret's labours, a report to which the names of Descamps, Portal, Hallé, and Cuvier are attached.

"The surface of the skin on which the cupping-glass is applied tends to supply the *vacuum* formed, and, as it swells, is raised into the cavity of the glass. The vessels and the *areolæ* of the subcutaneous cellular tissue dilate at the same time, and draw into the canals and expanded spaces a greater quantity of liquids, and these liquids are withdrawn in a circle (de proche en proche) from the neighbouring parts." (p. 113.)

M. Hallé asks the question, to what extent does the influence of cupping-glasses extend? We scarcely need remark that no distinct answer is given to this question. M. Gondret's opinion of the extent is tolerably wide, since in a case of affection of the heart, he applies his instruments round the pelvis, between the nates and on the upper part of the thighs. M. Hallé in a similar case places his, we think more rationally, between the shoulders. The results appear to confirm the predilection of M. Hallé, for in his case a cure is effected, in that of M. Gondret, relief.

The proceeding adopted by this latter gentleman was evidently founded on a doctrine to which we have already had occasion to advert. It is one of great antiquity and still prevails in France. According to this doctrine there are especial sympathies between some points of the surface and certain organs when in a state of disease, the sympathizing points not being those in the vicinity of the suffering organ but at some distance. Thus the integument of the inferior extremities is the situation selected for counter-irritation when the brain is the suffering organ; when the lung is so, it is that of the inner part of the arm. Barthez held the opinion that the sympathy existed especially with the surface of that perpendicular half of the body, right or left, in which the suffering organ was situated.* The British practice, as is well known, is in general to select

* Mémoires sur le Traitement méthodique des Fluxions qui sont les Elémens essentiels dans divers genres des Maladies; (Mém. de la Societé Méd. d'Emulation, t. ii.)

a portion of the surface lying over or closely bordering on the organ affected. The question between British and continental practice is one, in the language of the humoral pathology, between derivation and revulsion, and we own that we have never discovered any justifiable ground for the preference shown by our continental neighbours for the latter principle. Those peculiar remote sympathies, such as that between the legs and the brain, have ever appeared to us to be rather assumed than proved, rather the result of fancy than observation, and we have not always found their advocates in perfect accordance on the sympathising points. Were we to express the result of our own observation on these sympathies in general, we should say that they manifest themselves most decidedly between organs and the portion of the surface lying over them. We think this is abundantly exemplified in the applications of counter-irritants to relieve pain of the head, chest, bowels, and along the course of the spine.

VI. Before taking leave of the subject we think it right to caution the reader against expecting more from counter-irritation than it is able to accomplish. The cases are rare in which we should consider it more than subsidiary to internal medicine and properly regulated diet. A degree of exaggeration often occurs in works like those before us; but readers in general, being aware that the effect of the assiduous cultivation of any branch of science or study is to impart very high ideas of its importance, will know how to make the requisite deductions from statements in some degree overcharged, provided they do not transcend the ordinary level of such exaggeration. The following passage, however, does transcend this level, and we regard it as calculated to convey very deceptive ideas indeed, particularly to the class to which the work is especially addressed, of the power of external medication.

"On looking at these lists, it cannot be denied that the resources which the endermic physician, or he who treats diseases by external applications to the skin might command, are not inferior to those of the ordinary physician who relies principally on external [internal] remedies. Those resources or agents for internal [external] application would afford him the means of producing on the human body three several and successive degrees of artificial counter-irritation, by simply attending to any existing difference in the respective energies of the agents employed, or to the manner and length of time of their employment; or finally, to the various modes of preparing those agents for use." (*Granville*, p. 38.)

Through the haze of a very blundering phraseology, we can yet discern enough of the sense or rather nonsense of this passage to learn that the author places the power of external medication on an equality with that of internal; and this he does, because by outward remedies three several and successive degrees of counter-irritation may be induced. Is all we are capable of effecting by internal medicines—our various evacuants, our mercury, our antimony, our narcotics, our tonics, &c.,—but equal to three degrees of cutaneous irritation? If we look to the solvent and absorbent powers of the digestive canal, its acute sensibilities, and its wide and prompt sympathies, no judicious physician can hesitate to give it a general preference as a *medium* of producing a prompt and powerful action on the constitution over the cutaneous surface, even when we do not merely irritate the latter, but make it besides a vehicle for introducing powerful medicine into the system. In acute disease, threatening life, counter-irritation of the surface deserves confidence only as an auxiliary not as a principal; and the instances are rare in which the endermic method is adopted for powerfully medicating the constitution, and these instances are the exception not the rule. To the former of these allegations, the only one, be it remarked, involved directly in the question between Dr. Granville and ourselves, this gentleman may object his own case of pericarditis. This case we should have quoted in full, but it occupies five pages, and we are compelled to refer to page 297 of his volume, where the reader will find it. Dr. Granville, after being exposed when heated to a draught of cold air, feels ill on awaking in the morning, with pain in the thorax, "extending from the front to the back, through the heart, and down the left arm," and very irregular pulse. Medical assistance was sent for, but there was some delay and "the case must soon have become desperate." In three quarters of an hour, however, a young gentleman arrives and draws three cupfuls of blood. The pain still continues. A strong ammoniated embrocation is applied over the pained parts, and is kept on "until they become red hot, and burn vigorously." "In proportion as these effects were produced, the inward pain or stab-like feeling decreased, until at last it completely subsided and passed away." In an hour Dr. G. dressed, "and drove out in the carriage to his usual avocations for the rest of the day, without the slightest inconvenience. The pain never returned." Knowing that the application of the lotion should seldom last longer than from one to six or eight minutes, for Dr. Granville tells us so (see page 82 of his volume), and allowing ten minutes for the bleeding, an ample allowance, we find that pericarditis, one of the most formidable diseases to which the human frame is liable, was completely cured within twenty minutes! Every practical physician, every sound pathologist will at once declare that there is some mistake in this prompt cure of pericarditis, or, on the strength of one case of Dr. Granville's, they must abandon principles deduced from the observation of thousands of examples. Were we asked to give a name to this disease so rapidly removed, we should call it a combination of neuralgia and fright. We beg to remark that we do not consider ourselves as impeaching Dr. Granville's practical skill, in saying that he has erred in the diagnosis *of his own case.*

VII. The office of expressing our opinion of these works in any respect not already noticed will not detain us long.

1. Dr. Epps's little work we should not have noticed at all, it having been before the public for several years, but from its direct connexion, in subject, with the other more recent publications. It is written in a modest and unpretending spirit and in a clear and plain style, though with an occasional touch of quaintness. We recommend it to the attention of our readers not merely on account of these qualities, estimable as they are, but, moreover, for its good faith, earnestness, and the value of the information it imparts. We have already recorded our dissent from one of his principles of action or rather inaction, conceiving that he has occasionally inculcated rather a cowardly acquiescence in disease than a scientific correction of it, and we would say, too, that he attributes more preventive and curative power to issues than we have generally found them to manifest; still our general opinion of the work is very favorable.

2. We have already had occasion to intimate that counter-irritation and the various modes of effecting it have been more the objects of systematic study in France than in this country. This is no evidence that in the former country therapeutics have been more successfully cultivated than here. It rather appears to us conclusive of the opposite position; for where acute disease is most successfully combated, there will be found the fewest chronic ailments for the operation of heated iron and other counter-irritants; and many a triumph has been afforded to such means, which English practice, especially the judicious use of mercury, would have forestalled. However this may be, it is much to the credit of M. Gondret that, writing on a subject so much cultivated in France as to have become almost trite, his memoirs to the Royal Academy of Sciences, of which his volume may be regarded as the collective republication, should have drawn forth unqualified approbation from the commissioners of this distinguished body, and especially so, that one of these approving commissioners, M. Percy, should have been previously himself very much distinguished in the same field of investigation.* That in the present day any one in France could invest such matters as actual cautery and cupping glasses with interest, is abundant proof that there is something novel and consequently interesting in M. Gondret's application of these remedies, and that he has pursued the subject with zeal. This is the secret of his success and of the approbation he has received from high quarters: his heart is in his subject, and he consequently labours it with effect.

3. In conclusion we shall remark, that there are two classes of writers, those who write because they have something to say, and those who do so because they wish to say something. Fulness of knowledge and of thought is the inspiring genius of the one class; vanity and the love of display is the demon of the other. M. Gondret belongs to the former class; whilst Dr. Granville's present work places *him*, beyond all question, in the latter; and with this brief notice we finally dismiss it to that oblivion which (we hope) soon awaits it.†

* See his Pyrotechnie Chirurgicale, ou l'Art d'appliquer le Feu, and the articles *Moxibustion* and *Seton* in the Dict. des Sciences Médicales.

† Since the preceding article was printed, Dr. Granville has published, in the Lancet of October 27th, the formulæ for the preparation of his lotions. These we shall insert in Part Third of the present Number. We hope the reasons advanced by Dr. G., in this communication, for his having deferred to make known the composition of his lotions sooner, are satisfactory to his own feelings. We take no credit to ourselves for having contributed to this event by the strictures in our last Number; and we must say that we have found nothing in his recent communication that makes us at all regret having assumed the tone which characterizes the former article, and also the present, as far as Dr. Granville is concerned.—ED.

